# A global review of national influenza immunization policies: Analysis of the 2014 WHO/UNICEF Joint Reporting Form on immunization

**DOI:** 10.1016/j.vaccine.2016.07.045

**Published:** 2016-10-26

**Authors:** Justin R. Ortiz, Marc Perut, Laure Dumolard, Pushpa Ranjan Wijesinghe, Pernille Jorgensen, Alba Maria Ropero, M. Carolina Danovaro-Holliday, James D. Heffelfinger, Carol Tevi-Benissan, Nadia A. Teleb, Philipp Lambach, Joachim Hombach

**Affiliations:** a Department of Immunization, Vaccines and Biologicals, World Health Organization, Geneva, Switzerland; b Immunization and Vaccine Development, World Health Organization (WHO), South-East Asia Regional Office, New Delhi, India; c Division of Communicable Diseases and Health Security, World Health Organization (WHO) Regional Office for Europe, Copenhagen, Denmark; d Immunization Unit, Pan American Health Organization, Washington, DC, United States; e Expanded Programme on Immunization, World Health Organization (WHO) Regional Office for the Western Pacific, Manila, Philippines; f Immunization and Vaccine Development, World Health Organization, Regional Office for Africa, Brazzaville, People’s Republic of Congo; g Vaccine Preventable Diseases, World Health Organization (WHO) Regional Office for the Eastern Mediterranean, Cairo, Egypt

**Keywords:** Influenza vaccines, Immunization programmes, World Health Organization, Vaccination, Joint Reporting Form

## Abstract

**Introduction:**

The WHO recommends annual influenza vaccination to prevent influenza illness in high-risk groups. Little is known about national influenza immunization policies globally.

**Material and Methods:**

The 2014 WHO/UNICEF Joint Reporting Form (JRF) on Immunization was adapted to capture data on influenza immunization policies. We combined this dataset with additional JRF information on new vaccine introductions and strength of immunization programmes, as well as publicly available data on country economic status. Data from countries that did not complete the JRF were sought through additional sources. We described data on country influenza immunization policies and used bivariate analyses to identify factors associated with having such policies.

**Results:**

Of 194 WHO Member States, 115 (59%) reported having a national influenza immunization policy in 2014. Among countries with a national policy, programmes target specific WHO-defined risk groups, including pregnant women (42%), young children (28%), adults with chronic illnesses (46%), the elderly (45%), and health care workers (47%). The Americas, Europe, and Western Pacific were the WHO regions that had the highest percentages of countries reporting that they had national influenza immunization policies. Compared to countries without policies, countries with policies were significantly more likely to have the following characteristics: to be high or upper middle income (*p* < 0.0001); to have introduced birth dose hepatitis B virus vaccine (*p* < 0.0001), pneumococcal conjugate vaccine (*p* = 0.032), or human papilloma virus vaccine (*p* = 0.002); to have achieved global goals for diphtheria-tetanus-pertussis vaccine coverage (*p* < 0.0001); and to have a functioning National Immunization Technical Advisory Group (*p* < 0.0001).

**Conclusions:**

The 2014 revision of the JRF permitted a global assessment of national influenza immunization policies. The 59% of countries reporting that they had policies are wealthier, use more new or under-utilized vaccines, and have stronger immunization systems. Addressing disparities in public health resources and strengthening immunization systems may facilitate influenza vaccine introduction and use.

## 1 Introduction

The Global Action Plan for Influenza Vaccines (GAP^1^) was launched by the World Health Organization (WHO) in 2006 to reduce the global shortage of seasonal and pandemic influenza vaccines [1]. A major goal of GAP is to increase seasonal influenza vaccine use globally. Addressing this goal would prevent severe influenza illness, strengthen health systems within countries to better respond to influenza pandemics, and encourage the vaccine industry to develop greater influenza vaccine manufacturing capacity [1]. Since the launch of GAP, regional influenza vaccine manufacturing capacity has expanded and overall global production capacity has increased [2], [3], [4], [5]. As of 2015, global production capacity for a pandemic influenza vaccine is estimated to be around 6.372 billion doses per year [5]. While this estimate represents a large increase from the most recent assessment, production capacity is still far from the GAP goals of being able to provide two doses of vaccine to 70% of the global population within 6 months [1].

The 2012 WHO position paper on influenza vaccines notes that influenza causes considerable morbidity worldwide and that countries should consider implementing influenza vaccine programmes as national capacities and resources allow [6]. WHO recommends that countries consider immunizing high-risk groups against influenza; these groups include pregnant women, young children 6–59 months, adults with specific chronic illnesses, persons at least 65 years, and health care workers [6]. As of the 2012 position paper, WHO recommends that countries initiating or expanding influenza immunization programmes should prioritize pregnant women, while other risk groups are not ranked by priority [6]. There are limited published data about the presence or nature of national influenza immunization policies in many countries. We reviewed data reported by Member States to WHO and UNICEF to evaluate national influenza immunization priorities and use to inform efforts to meet WHO influenza immunization goals [1], [7].

## 2 Materials and methods

We adapted the WHO/UNICEF Joint Reporting Form on Immunization (JRF) to include questions on national influenza immunization policies and use. Since 1998, the JRF is completed annually by Ministries of Health of WHO Member States [8]. The JRF is a monitoring and evaluation tool that collects national administrative information regarding estimates of immunization coverage, reported cases of vaccine-preventable diseases, immunization schedules, vaccination campaigns, as well as indicators of immunization system performance and financing [9]. Most WHO Member States report immunization data annually in the JRF. Prior to 2014, the JRF collected information about influenza vaccine schedules, distribution, and coverage. At that time, WHO adapted the influenza vaccine JRF questions to focus on presence of seasonal influenza immunization policies during the preceding year, 2014 (see Table 1). Collected data included whether the country had a national seasonal influenza vaccination policy, the groups targeted for vaccination by the policy, the influenza vaccine formulation used (northern or southern hemisphere), and the number of influenza vaccine doses distributed. Vaccine distribution data are collected as an approximate measure of vaccine use, since data on doses administered are not available in many countries. Influenza vaccine coverage among persons with chronic illnesses and among the elderly are also collected to monitor progress toward World Health Assembly resolution WHA56.19 goals that countries with influenza vaccine programmes immunize ⩾ 75% of persons in these groups [7]. The entire JRF undergoes standard data checks at national, regional, and global levels to improve data quality and consistency. We reviewed the specific JRF influenza vaccine data. We used regional surveys from the Americas, Europe, and the Western Pacific to determine the presence of immunization policies for some countries that did not report influenza data on the JRF [10], [11], [12], [13], [14]. We did not extract vaccine coverage, formulation, or distribution data from non-JRF sources. We used the 2014 JRF database that was available as of 15 July 2016.

Next, we identified additional Member-State-specific data (from 2014 except where noted) that were relevant to immunization programmes (Table 4). As a measure of country economic status, we used 2013 World Bank data for country income category and health expenditure per capita [15]. As low-resource countries often rely on financial support from Gavi, the Vaccine Alliance, for new vaccine introduction, we also used JRF data for country eligibility to receive such support (Gavi eligible) [16], [17]. To assess new vaccine introductions, we used JRF data indicating the inclusion of the following vaccines in national immunization schedules per WHO recommendations: birth dose hepatitis B vaccine (HBV), pneumococcal conjugate vaccine (PCV), human papilloma virus vaccine (HPV), and rotavirus vaccine [18], [19], [20]. To assess the general strength of routine immunization we used JRF data indicating whether countries had achieved Global Vaccine Action Plan (GVAP) goals of maternal and neonatal tetanus elimination (MNTE) (<1 case of neonatal tetanus per 1,000 live births in every district of a country), and we used the WHO UNICEF estimates of national immunization coverage to assess if countries had achieved global goals of diphtheria-tetanus-pertussis containing vaccine third dose coverage (DTP3) ⩾95% nationally [21], [22]. Finally, to assess the strength of a country to make decisions about vaccine interventions, we used JRF data to determine whether countries achieved the indicators defining well-functioning National Immunization Technical Advisory Groups (NITAGs) [21], [23]. We did not have data regarding whether a country participated in a well-functioning subregional Technical Advisory Group (TAG), which is recommended as an option for small countries. Country income category (high income/upper middle income vs. lower middle income/low income), new vaccine introduction, routine immunization, and NITAG covariates were defined as dichotomous variables for statistical analyses.

We used descriptive statistics to describe JRF responses globally among all 194 WHO Member States and within the six WHO regions: Africa (AFR), 47 countries; Americas (AMR), 35 countries; Eastern Mediterranean (EMR), 21 countries; Europe (EUR), 53 countries; South-East Asian (SEAR), 11 countries; and Western Pacific (WPR), 27 countries [24]. Our analysis excluded non-Member States and territories. We conducted bivariate analyses to compare covariate association with presence of a national influenza immunization policy using *χ*^2^ for categorical variables and Wilcoxon-Mann-Whitney test for non-parametric continuous variables. All statistical tests were two sided, and *p*-values < 0.05 were considered statistically significant. Missing data were excluded from analyses. We used Stata statistical software version 14.0 (StataCorp; College Station, TX, United States of America). As we used publicly available, national-level data collected for monitoring and evaluation of public health programmes, no human subjects approval was needed.

## 3 Results

Of 194 WHO Member States, 187 (96%) submitted a 2014 JRF to WHO and UNICEF and 162 (84%) provided data regarding influenza immunization policies in the JRF. Data were reported from each WHO region, including AFR (100%), AMR (91%), EMR (81%), EUR (74%), SEAR (100%), and WPR (63%). After review of other published sources [10], [11], [13], [14], we were able to identify influenza immunization policy information for 189 (97%) Member States.

**Fig. 1:**
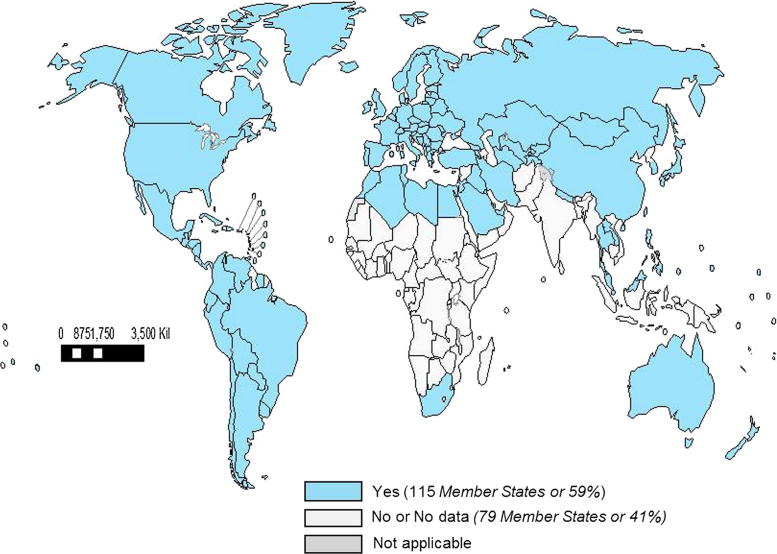
World Map with WHO Member States Reporting to Have a National Seasonal Influenza Vaccine Programme in 2014. Note: The boundaries and names shown and the designations used on this map do not imply the expression of any opinion whatsoever on the part of the World Health Organization concerning the legal status of any country, territory, city or area or of its authorities, or concerning the delimitation of its frontiers or boundaries. Dotted lines on maps represent approximate borderlines for which there may not yet be full agreement. ©WHO 2016. All rights reserved.

*Source*: WHO-UNICEF Joint Reporting Forms (JRFs) 2014. Map production: Immunization Vaccines and Biologicals, (IVB). World Health Organization. Date of slide: 15 July 2016.

**Fig. 2:**
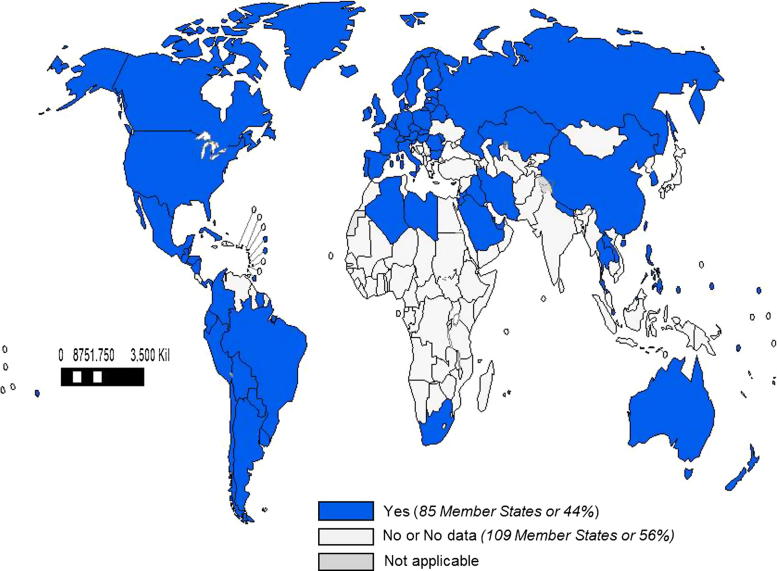
World Map with WHO Member States Reporting to Have a National Seasonal Influenza Vaccine Programme that Targets Either All Persons > 6 Months or Pregnant Women in 2014. Note: The boundaries and names shown and the designations used on this map do not imply the expression of any opinion whatsoever on the part of the World Health Organization concerning the legal status of any country, territory, city or area or of its authorities, or concerning the delimitation of its frontiers or boundaries. Dotted lines on maps represent approximate borderlines for which there may not yet be full agreement. ©WHO 2016. All rights reserved.

*Source*: WHO-UNICEF Joint Reporting Forms (JRFs) 2014. Map production: Immunization Vaccines and Biologicals, (IVB). World Health Organization. Date of slide: 15 July 2016.

Of the 194 WHO Member States, 115 (59%) countries reported having a national influenza immunization policy (Fig. 1, Table 2). No country reported that it had a national policy to not use influenza vaccine. Among the countries with a national policy, immunization programmes targeted specific WHO-defined risk groups, including pregnant women (42%) (Fig. 2), young children (28%), adults with chronic illnesses (46%), the elderly (45%), and health care workers (47%). Fifteen percent of countries that reported national policies targeted all persons > 6 months for immunization with influenza vaccine. Among countries with policies, many targeted additional groups for immunization, including children with chronic illnesses (if all children were not already targeted to receive vaccine) (15%) and Hajj or other travellers (18%). There were national influenza immunization policies in countries classified as high income (92%), upper middle income (79%), lower middle income (37%), and low income (3%). The number and percentage of countries in WHO regions that reported having influenza immunization policies are: 3 (6%) in AFR, 31 (88%) in AMR, 12 (57%) in EMR, 51 (96%) in EUR, 2 (18%) in SEAR, and 16 (59%) in WPR (Table 2). The proportion of countries with policies targeting specific risk groups was similar among regions. Of the 16 countries that manufacture influenza vaccines [5], 14 (88%) reported having a national influenza immunization policy.

Eighty-nine (46%) countries provided information on vaccine formulation used, and of these, 60 (67%) used the northern hemisphere formulation, 19 (21%) used the southern hemisphere formulation, and 10 (11%) used both formulations. Sixty-six (34%) countries provided vaccine distribution data. These countries reported distributing of 230,258,405 doses of seasonal influenza vaccine, or around 37% of the estimated 620 million doses produced annually [4]. Vaccine doses were distributed in EUR (39%), AMR (29%), WPR (28%), SEAR (1%), AFR (1%), and EMR (1%). Data were missing from 128 countries (56%), including countries that reported elsewhere to have high influenza vaccine distribution rates [25].

A small number of countries reported influenza vaccine coverage that meets World Health Assembly resolution goals to immunize ⩾ 75% of persons with chronic underlying diseases and the elderly. Twenty-five countries (13%) reported influenza immunization rates in persons with chronic diseases; among these, coverage ranged from 4% to 100%, and 5 countries (3%) reported achieving coverage ⩾ 75%. Thirty-seven countries (19%) reported influenza immunization rates in the elderly; among these, coverage ranged from 1% to 100%, and only 2 countries (1%) reported achieving coverage ⩾ 75%.

Countries reporting national influenza immunization policies were significantly more likely to be high income or upper middle income classification (83% vs 20%, *p* < 0.0001) and to be ineligible for Gavi funding support (3% vs 57%, *p* < 0.0001). Countries with policies also had significantly higher median per capita health expenditures than those without such policies (664 USD vs 82 USD, *p* < 0.0001) (Table 2). In addition, countries with policies were significantly more likely to have introduced birth dose HBV vaccine (68% vs 37%, *p* < 0.0001), introduced PCV vaccine (70% vs. 54%, *p* = 0.032), introduced HPV vaccine (49% vs. 9%, *p* = 0.002), and to have a functioning NITAG (53% vs. 22%, *p* < 0.0001). Introduction of rotavirus vaccine was not significantly associated with influenza immunization policies. Countries with policies were more likely to have eliminated neonatal tetanus (98% vs 72%, *p* < 0.0001) and to have achieved DTP3 coverage ⩾ 95% nationally (83% vs 42%, *p* < 0.0001). Only four countries with influenza immunization policies were Gavi-eligible (Table 3); of these, two had functioning NITAGs, three had introduced birth dose HBV vaccine, one had introduced PCV vaccine, one had introduced HPV vaccine as a demonstration project, and none had introduced rotavirus vaccine.

## 4 Discussion

The 2014 revision of the WHO/UNICEF JRF on Immunization permitted a global summary of national influenza immunization policies. Such policies were reported by 59% of WHO Member States, including countries from all WHO regions and all World Bank income categories. Our report shows increased global influenza immunization policy development. A 2006 global survey identified 74 countries and territories with seasonal influenza immunization policies [26]. That survey of countries and territories was conducted by WHO as part of the 2006 GAP launch and achieved a 70% response rate. We identified 115 WHO Member States with influenza immunization policies in 2014. Since 2006, policy development was seen in each WHO region, and the countries targeting pregnant women for influenza vaccine receipt has increased from around 15 in 2006 to 81 in 2014. Annual reporting by countries with the JRF will facilitate continued monitoring of progress of influenza immunization policy development.

This report highlights that work must still be done to ensure equitable access of influenza vaccines globally. The regions with the highest percentages of Member States with influenza immunization policies (AMR, EUR, and WPR) reported distributing 97% of all influenza vaccines. While we had low response rates for influenza vaccine distribution, the proportion of vaccine distribution by WHO regions are similar to previous reports using more robust distribution data by the International Federation of Pharmaceutical Manufacturers & Associations [25]. Further, 42% of countries reported having specific programmes for immunization of pregnant women, a risk group prioritized for influenza vaccine receipt by WHO beginning in 2012. Finally, few countries reported meeting World Health Assembly goals to immunize ⩾ 75% of elderly persons and ⩾ 75% of persons with chronic illnesses [7], though other data sources indicate more countries have achieved these goals [10], [11].

Findings from this survey may facilitate the initiation or expansion of influenza vaccine programmes globally. Countries in higher World Bank income categories were significantly more likely to have influenza immunization policies. Other factors associated with presence of influenza immunization policies included experience introducing other new and under-utilized vaccines such as birth dose HBV vaccine, PCV, and HPV. Countries with influenza immunization policies had strong routine immunization programmes as measured by DTP3 coverage as well as by achieving elimination of maternal and neonatal tetanus. Many countries without influenza policies did not have functional NITAGs. Strengthening NITAGs to support review of evidence to make vaccine recommendations is critical for country decision making. Lastly, the lack of financing mechanisms to support influenza vaccine introduction in low-resource countries will pose a major challenge. Knowledge of the factors that influenced the development of influenza immunization policies would be of high value for efforts to increase influenza vaccine use globally.

The report should be interpreted in the context of several limitations. Data were collected from the first year that influenza immunization policy questions were asked in the JRF. WHO experience with the JRF is that completeness and accuracy of reported data increases over time with increased familiarity with the form. The JRF had a substantial amount of missing data on influenza vaccines, particularly regarding vaccine distribution and coverage estimates. Some countries that have made progress achieving influenza vaccine coverage goals or distributing influenza vaccines did not provide these data in the JRF. Improving influenza vaccination monitoring and reporting will be vital if the JRF is to be used to monitor progress in influenza vaccine use prospectively. Additionally, some countries, mostly high income countries from the Americas and Europe, did not report influenza vaccine information, requiring us to seek relevant policy data through reviews of public information. Finally, the presence of an influenza immunization policy does not necessarily correlate with substantial vaccine use.

The JRF is an efficient and very useful tool to prospectively monitor annual progress toward implementation and equitable use of influenza vaccines globally. WHO recommends annual vaccination of high-risk groups to prevent complications of influenza virus infection, however this report demonstrates that many countries do not yet have national influenza immunization policies. Countries without influenza immunization policies tend to have fewer resources, use fewer new or under-utilized vaccines, and have weaker routine immunization systems. Addressing these challenges may facilitate influenza vaccine introduction and use.

## Conflicts of interest

The authors report no conflicts of interest.

## Disclaimer

Authors of this study are employees of the World Health Organization. The authors alone are responsible for the views expressed in this publication and they do not necessarily represent the decisions, policy, or views of the World Health Organization.

## Funding

The authors would like to acknowledge the contributions of the Centers for Disease Control and Prevention (CDC), which provides financial support to the World Health Organization Initiative for Vaccine Research (U50 CK000431).

## Figures and Tables

**Table 1:** 2014 WHO/UNICEF Joint Reporting Form (JRF) on immunization influenza vaccine questions.

Does the country have a national seasonal influenza vaccination policy?
Which risk groups if any, are recommended for seasonal influenza vaccination? • Children (if yes, specify age range in comment field) • Older persons (if yes, specify age range in comment field) • Chronic illness (paediatric) • Chronic illness (adult) • Pregnant women • Health care workers • Hajj or other travellers • Any other risk group (if yes, please specify in the comment field) • All persons > 6 months are recommended to receive vaccine • No groups are specified for influenza vaccine receipt
How many doses of seasonal influenza vaccine were distributed in 2014?
What seasonal influenza vaccine formulation was used in 2014?
What was the percentage (%) of elderly persons immunized against influenza in 2014?
What was the percentage (%) of persons with underlying disease immunized against influenza in 2014?

**Table 2:** WHO Member States with influenza immunization policies in 2014, by WHO region.

Region	Member States	Has national influenza immunization policy *n*,%	Targets children *n*,%	Targets adults with chronic illness *n*,%	Targets pregnant women *n*,%	Targets health care workers *n*,%	Targets elderly *n*,%	Targets other groups *n*,%
Africa (AFR)	47	3 (6%)	2 (4%)	3 (6%)	3 (6%)	3 (6%)	3 (6%)	2 (4%)
Americas (AMR)	35	31 (88%)	22 (63%)	20 (57%)	21 (58%)	23 (66%)	18 (51%)	14 (40%)
Eastern Mediterranean (EMR)	21	12 (57%)	6 (29%)	11 (52%)	8 (38%)	10 (48%)	9 (43%)	12 (57%)
Europe (EUR)	53	51 (96%)	12 (23%)	33 (63%)	34 (64%)	37 (70%)	39 (74%)	29 (55%)
South-East Asian (SEAR)	11	2 (18%)	2 (18%)	2 (18%)	2 (18%)	2 (18%)	2 (18%)	2 (18%)
Western Pacific (WPR)	27	16 (59%)	8 (30%)	12 (44%)	12 (44%)	15 (56%)	15 (56%)	8 (30%)
Worldwide	194	115 (59%)	54 (28%)	89 (46%)	81 (42%)	91 (47%)	87 (45%)	62 (32%)

*Notes*: Data source is 2014 WHO/UNICEF Joint Reporting Form (JRF) augmented by policy data from regional surveys [10], [11], [13], [14].

**Table 3:** WHO Member States with influenza immunization policies in 2014, by country income status.

Country income status	Member States	Has national influenza immunization policy *n*, %	Targets children *n*, %	Targets adults with chronic illness *n*, %	Targets pregnant women *n*, %	Targets health care workers *n*, %	Targets elderly *n*, %	Targets other groups *n*, %
Low and lower-middle income countries that are GAVI-eligible	49	4 (8%)	1 (2%)	2 (4%)	2 (4%)	2 (4%)	2 (4%)	1 (2%)

Low and lower-middle income countries that are not GAVI-eligible	34	16 (47%)	7 (21%)	10 (29%)	9 (26%)	14 (41%)	11 (32%)	8 (24%)

Upper middle income countries	52	41 (79%)	19 (37%)	28 (54%)	26 (50%)	30 (58%)	24 (46%)	16 (31%)

High income countries	59	54 (92%)	26 (44%)	49 (83%)	44 (75%)	45 (76%)	50 (85%)	37 (63%)

*Notes*: Data source is 2014 WHO/UNICEF Joint Reporting Form (JRF) augmented by policy data from regional surveys [10], [11], [13], [14].

**Table 4:** Comparison of WHO Member States with and without national influenza immunization policies in 2014.

	Countries with a national influenza immunization policy *n*,%	Countries without a national influenza immunization policy *n*,%
Total countries	115	79

Country wealth		
Median per capita health expenditure (USD$), (IQR)	664 (342, 2018)	82 (42, 178)
World Bank income category		
High income	54 (47%)	5 (6%)
Upper middle income	41 (36%)	11 (11%)
Lower middle income	19 (17%)	33 (42%)
Low income	1 (1%)	30 (38%)
Gavi-eligible	4 (3%)	45 (57%)

New and Under-utilized vaccine introduction		
Introduced birth dose hepatitis B vaccine (HBV)	78 (68%)	29 (37%)
Introduced pneumococcal conjugate vaccine (PCV)	80 (70%)	43 (54%)
Introduced human papilloma virus vaccine (HPV)	56 (49%)	7 (9%)
Introduced rotavirus vaccine	45 (39%)	30 (38%)

Strength of immunization systems		
Functioning National Immunization Technical Advisory Group	61 (53%)	17 (22%)
Eliminated maternal and neonatal tetanus	113 (98%)	57 (72%)
DTP3 coverage ⩾95% nationally	96 (83%)	33 (42%)

*Note*: Data on influenza immunization policies, functional NITAGs, maternal neonatal tetanus elimination, DTP3 coverage, and MCV2 coverage from 2014 WHO/UNICEF Joint Reporting Form (JRF) and augmented by policy data from regional surveys [10], [11], [13], [14]. Average per capita health expenditure and income category data are from World Bank.

## Footnotes

1 **Nonstandard abbreviations used in text:**

• AFR: WHO Africa Region
• AMR: WHO Region of the Americas
• DTP3: third dose of diphtheria-tetanus-pertussis vaccine
• EMR: WHO Eastern Mediterranean Region
• EUR: WHO Europe Region
• GAP: Global Action Plan for Influenza Vaccines
• JRF: WHO/UNICEF Joint Reporting Form on Immunization
• MNTE: maternal and neonatal tetanus elimination
• NITAG: National Immunization Technical Advisory Group
• SEAR: WHO South-East Asia Region
• UNICEF: United Nations Children’s Fund
• WPR: WHO Western Pacific Region
